# Direct observation of nucleation in the bulk of an opaque sample

**DOI:** 10.1038/srep42508

**Published:** 2017-02-14

**Authors:** Chaoling Xu, Yubin Zhang, Andrew Godfrey, Guilin Wu, Wenjun Liu, Jonathan Z. Tischler, Qing Liu, Dorte Juul Jensen

**Affiliations:** 1 Section for Materials Science and Advanced Characterization, Department of Wind Energy, Technical University of Denmark; 2 School of Materials Science and Engineering, Chongqing University, Chongqing 400044, China; 3 Key Laboratory of Advanced Materials (MoE), School of Material Science and Engineering, Tsinghua University, Beijing 100084, P.R. China; 4 Advanced Photon Source, Argonne National Laboratory, Argonne, Illinois 60439-4800, USA

## Abstract

Remarkably little is known about the physical phenomena leading to nucleation of new perfect crystals within deformed metals during annealing, in particular how and where volumes with nearly perfect lattices evolve from structures filled with dislocations, and how local variations at the micrometer length scale affect this nucleation process. We present here the first experimental measurements that relate directly nucleation of recrystallization to the local deformation microstructure in the bulk of a sample of cold rolled aluminum, further deformed locally by a hardness indentation. White beam differential aperture X-ray microscopy is used for the measurements, allowing us to map a selected gauge volume in the bulk of the sample in the deformed state, then anneal the sample and map the exact same gauge volume in the annealed state. It is found that nuclei develop at sites of high stored energy and they have crystallographic orientations from those present in the deformed state. Accordingly we suggest that for each nucleus the embryonic volume arises from a structural element contained within the voxels identified with the same orientation. Possible nucleation mechanisms are discussed and the growth potentials of the nuclei are also analyzed and discussed.

When deformed metals are annealed, recrystallization typically occurs, during which small almost perfect crystals called nuclei form and subsequently grow by boundary migration through the deformed matrix until the deformation microstructure is replaced^1^,^2^,^3^. The driving force for recrystallization is the energy stored in the deformed matrix in the form of crystal lattice defects such as vacancies and dislocations^1^,^3^. Considering that annealing and thus recrystallization is used in almost every metal forming process, it is surprising that very little is known about actual nucleation mechanisms and nucleation sites. Although it is well accepted that original grain boundaries and triple junction lines (where 3 grains meet) are preferential nucleation sites^4^, at which the nucleation mechanism may be strain induced boundary migration^5^,^6^, it is not known where, along the many kilometers of original grain boundaries and triple junction lines that are present in typical samples, nuclei will form. Large second phase particles are also known to stimulate nucleation^7^,^8^, but open questions are whether all particles stimulate nucleation, or only some of them, and if so which ones^9^,^10^? Finally, surface imperfections such as scratches can act to stimulate nucleation, but scratches are non-controllable^11^,^12^. Nucleation from all other sites, including a range of local heterogeneities, which may form the majority of events, is even less clear.

A key reason for the paucity of knowledge regarding nucleation of recrystallization is the lack of suitable experimental techniques for studying this process. Nuclei are few and small, which severely complicates any characterization. Moreover, they form in a so-far unpredictable way at sites in the bulk away from sample surfaces, and their formation can thus not be observed directly *in-situ* by light or electron microscopy. Static, “after the fact”, observations on the other hand, do not help to pinpoint nucleation mechanisms, as the deformed matrix leading to nucleation is consumed–a problem often referred to as that of “lost evidence”^13^. A consequence of this is that suggested nucleation mechanisms have not been verified by direct experimental measurements.

It has been a dream to follow directly nucleation in the bulk of deformed samples, and accordingly various attempts to investigate nucleation have already been performed using three dimensional X-ray diffraction (3DXRD)^14^,^15^. In each case, however, the spatial resolution of the measurements was insufficient to allow mapping of the microstructural subdivision of the deformed microstructure (the spatial resolution in these measurements was ≥4 μm). As such it could not be established in these studies which microstructural elements lead to nucleation.

In the present work, we use another synchrotron X-ray method that allows us to surmount these challenges such that we can resolve the deformation induced microstructural subdivision and directly observe nucleation in the bulk of a metal sample. We present results of an investigation where we first characterize in detail the deformed microstructure of a selected volume in 3D (x, y and z) non-destructively. The sample is then annealed in a controlled manner to allow a few nuclei to form within the initially characterized volume, and finally the 3D microstructure within the selected sample volume is characterized again. Accordingly it is possible to quantify precisely where in the deformed microstructure the nuclei are formed, and what crystallographic orientation relationships exist between each nucleus and its parent deformation structural “element”. This further allows for an analysis of which microstructural elements are potential nucleation sites.

The experimental method used is white-beam differential-aperture X-ray microscopy (DAXM)^16^. As described in the Methods Section, spatially resolved DAXM experiments were conducted to determine the crystallographic orientations in 3D of the selected sample before and after annealing with a high spatial resolution of 1.5 × 1.5 × 1.5 μm^3^ and an angular resolution of 0.01°. The measurements are quite time consuming: 65 hours are needed to map a gauge volume of 243 × 79.5 × 76.5 μm^3^ at this spatial resolution. It is thus critical to choose a sample for which there is a high probability for the formation of just few nuclei within the selected gauge volume, as if no nucleation occurs, or nucleation occurs but the nuclei grow outside the mapped volume, the entire experiment is vain. To achieve such control of the nucleation lightly cold rolled high purity aluminum, further deformed locally by a Vickers hardness indentation has been chosen. The hardness indentation is needed to allow some control over the general volume in which nucleation is expected to occur. Although hardness indentations are known to be preferential nucleation sites, it is also the case that not every indentation will stimulate nucleation. Therefore in preparation for this experiment, several hundred indentations were characterized, considering two different loads, in 15 grains of different orientation, for samples of pure aluminum rolled to two different reductions in thickness^17^,^18^ and the optimal condition was chosen. At the same time the appropriate annealing conditions to allow the formation of just a few nuclei were also established. Besides allowing some control of the potential nucleation site, nucleation in a sample deformed by hardness indentations is interesting as it allows comparison with the literature on the complex deformation zones with strong gradients developed around indentations, based on both experiments and simulations^19^,^20^. The light cold rolling of the sample was chosen to provide sufficient stored energy for the growth of the nuclei. Aluminum was chosen because it has relatively simple crystal symmetry (fcc) and yet its recrystallization behaviour is typical for many metals used industrially.

## Results and Discussion

**Figure 1:**
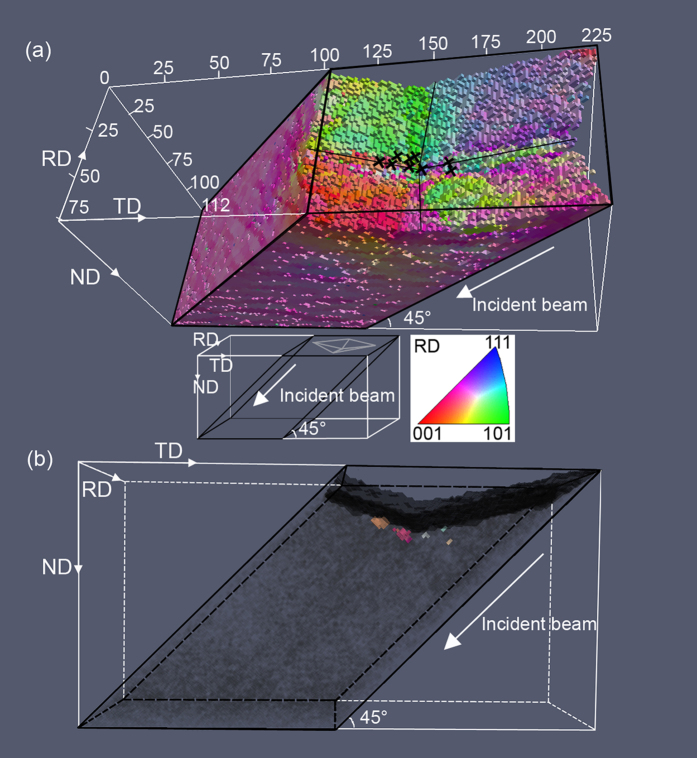
Overview of the mapped deformed volume and the embryonic volumes of the nuclei. (**a**) is a three-dimensional (3D) map of the spatial distribution of crystallographic orientations within the sample in the deformed state near the indentation tip. The colors show the crystallographic orientation of the rolling direction (RD) within each voxel according to the color legend shown. The diagonal lines of the indent are marked by thin black lines. The black crosses in the 3D map indicate the positions of the embryonic volumes that develop into nuclei upon annealing. The figure shows that all nuclei appear near the tip, and along the diagonal lines, of the Vickers hardness indentation. The relationship between the mapped sample volume and sample (RD, TD and ND) coordinate system is shown in the inset, where the grey lines at the top of the sample indicate the orientation of the hardness indentation in relation to the sample coordinate system. It should be noted that the mapped volume does not include the top part of the indent, which in total is ~26.3 μm in depth and ~184 μm in diagonal length. (**b**) is a side view showing the positions of the embryonic volumes of the nuclei. The voxels colored in dark grey show the indented surface of the sample, identified by locating the voxels immediately adjacent to those giving a diffracted signal from the sample, i.e. the dark grey voxels are the most precise surface identification that can be made. The light grey voxels are the whole mapped volume. The characterized volume is marked by thick black lines. The incident X-ray beam direction is marked by white arrows. The figure shows that the nuclei form very near the indented surface.

The deformed microstructure in the gauge volume around the indentation is shown in Fig. 1a. It can be seen that the Vickers hardness indentation created a deformation zone, where sharp changes in orientations are seen in particular along the symmetry lines of the indentation. This is in agreement with earlier destructive experimental characterization and simulations^17^,^19^,^20^. After annealing at 275 °C for 10 min, nucleation occurs in the gauge volume of the sample in several locations near the indentation tip. In total, 12 nuclei were observed within the gauge volume. Among these nuclei, two were twins, one of which grew out of the gauge volume. Another nucleus grew to a very large size. These three nuclei are not analyzed further in the following, as it is not possible to identify where exactly these nuclei formed. The remaining 9 nuclei are marked in Fig. 1a, and in 3D in Fig. 1b. It can be seen that all 9 nuclei are near the indentation tip, and more specifically along the symmetry lines in the indentation zone formed by the ridges of the diamond shaped indenter.

**Figure 2:**
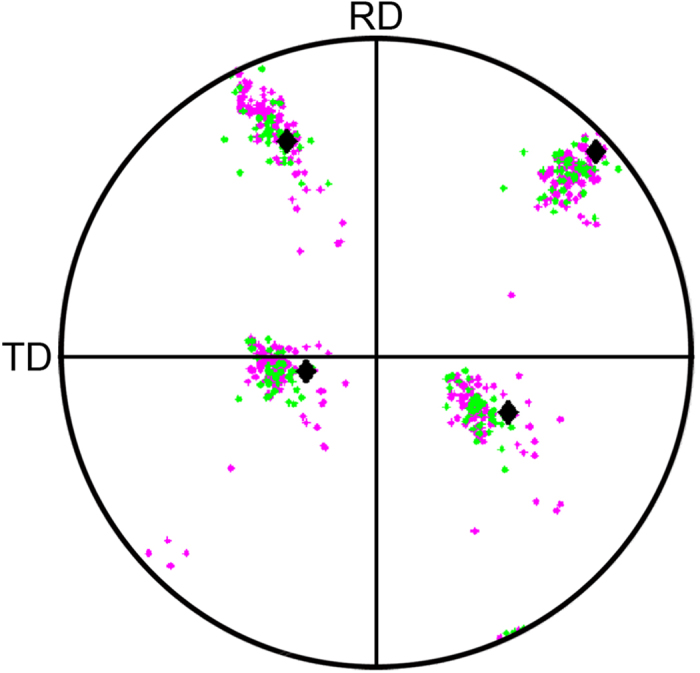
{111} pole figure showing the orientation relationship between nucleus No. 3 and the matrix in the corresponding volume in the deformed state before annealing. The nucleus orientation is shown by large black diamond shaped markers. Magenta small diamond shaped markers show the orientations in the deformed state for voxels identified as belonging to the nucleus after annealing, and green small diamond shaped markers show voxels belonging to the “cloud” around the nucleus volume, included to allow for the possible small sample misalignment when remounting the sample after the annealing. The figure shows that nucleus No. 3 has an orientation within the scatter of orientations in the deformed state at the nucleation site.

The orientation relationship between each nucleus and the matrix in which it forms is analyzed by plotting a pole figure showing the orientation of the nucleus and the orientations present in the corresponding volume before the sample was annealed, and which was consumed during the formation of the nucleus. An example is shown in Fig. 2. The orientation of the nucleus is shown by a diamond and the orientations within the deformed matrix in the consumed volume are shown by magenta dots. To allow for the slight uncertainty in sample position when remounting the sample after furnace annealing, voxels in a “cloud” of approximately 1.5 μm around the nucleus are included in the analysis. The orientations within this cloud are shown by green dots in Fig. 2. The data show that the nucleus orientation is within the distribution of orientations present in the deformed matrix in the consumed volume. This is the case for all of the 9 nuclei analyzed in the present study (see supplementary Fig. S1). In one case, the nucleus orientation is on the outskirts of the orientation spread in the deformed matrix. No correlation is found between nucleus size and its orientation within the orientational scatter of the consumed deformation microstructure. For example, the nucleus with orientation towards the outskirts of the deformed orientational scatter is neither bigger nor smaller than nuclei with orientations well within the orientational scatter.

**Figure 3:**
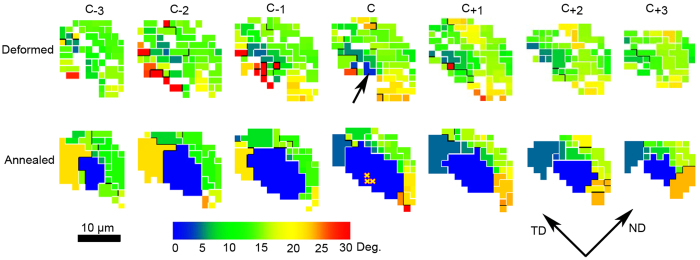
Sections through the 3D volume in the deformed and the annealed state near nucleus No. 3. The successive sections are 1.5 μm apart along the rolling direction. The nucleus is shown in blue and all other voxels are colored according to the misorientation angle to the nucleus orientation. The white and black lines show misorientations in the range 2–15° and above 15°, respectively. TD and ND the represent transverse direction and the normal direction of the sample, respectively. By inspecting the orientations in the deformed state, it is clear that 3 voxels, marked by an arrow, have the same orientations as the nucleus. All other voxels have different orientations. It is thus suggested that these three voxels represent the embryonic volume of nucleus No. 3 (also marked by yellow crosses in the sections showing the annealed state).

The spatial resolution of the present DAXM data also allow determination of where exactly the nuclei form in the consumed volume, i.e. identification of the nucleation sites, by comparing the orientations of all the voxels in the consumed deformation volumes with the orientations of the nuclei. As described above we also in this analysis include “a cloud” around each nucleus to allow for possible slight misalignments during sample remounting. An example is shown in Fig. 3. Here the microstructure is visualized slice-by-slice in the deformed and annealed state. The nucleus is shown in blue. The color of each voxel in the map of the deformed state is chosen to show its misorientation to the nucleus. It can be seen that in the middle slice of the deformed microstructure, 3 voxels (marked by an arrow) have orientations similar (<2°) to the nucleus. Nowhere else are such identical orientations found. It is thus reasonable to assume that the voxels marked by an arrow contain, or represent, the embryonic volume for this nucleus. This is substantiated by the fact that 7 of the other 9 nuclei follow the same pattern, with only two or three voxels in the deformed state having orientations similar (<2°) to that of the nucleus, such that an embryonic-containing volume of either 6.75 or 10.13 μm^3^ can be defined. For the other two cases a somewhat larger number of voxels with similar orientations to the nuclei are found (12 and 35), although even in these cases this still represents a small fraction (less than 1/10^th^) of the nuclei volume.

**Figure 4:**
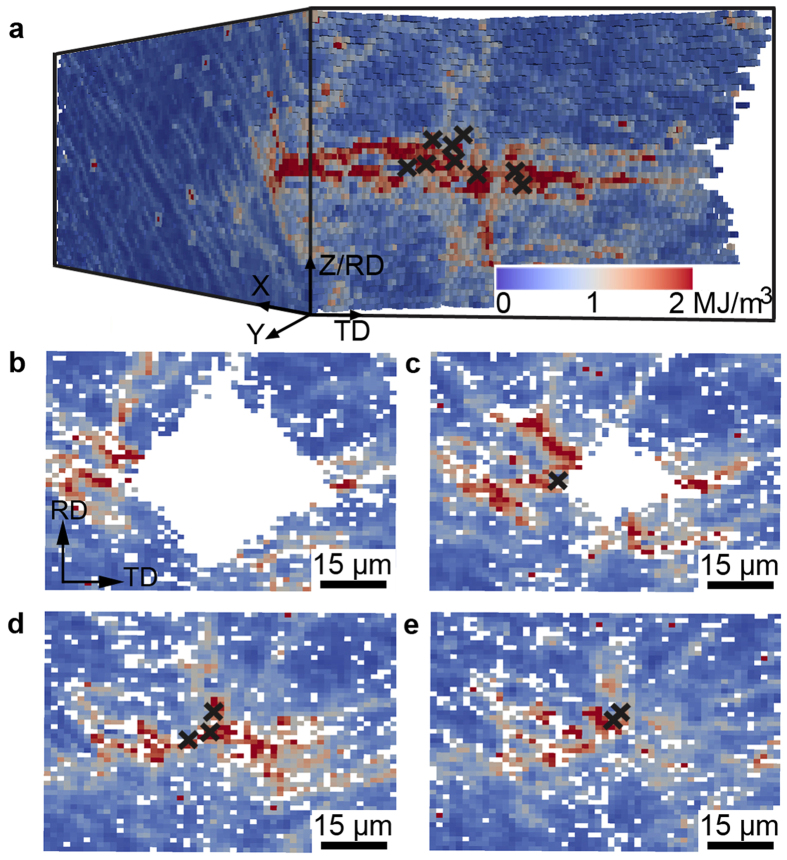
Distribution of stored energy in the deformed state. (**a**) is a 3D map of the stored energy (magnitude indicated by the color legend). The crosses mark the positions of the identical embryonic volumes. (**b**,**c**,**d** and **e**) show sections near the indentation tip: (**b**) 8.5 μm above, (**c**) 4.3 μm above, (**d**) at exactly at, and (**e**) 3.2 μm below the tip.

The above analysis leads to a precise pinpointing of nucleation sites, which are marked in Figs 1a and 4. It is found that all nuclei form very near the indented surface (within 4.5 μm from the surface) and within a zone not further than 15.0 μm away from the indentation tip. To understand why these sites are the active ones, the local stored energy contained in the dislocation boundaries in the deformed matrix is calculated using the kernel average misorientation (KAM) method suggested in ref. 21. For the calculation of the KAM value at each voxel, a low cut-off angle of 0.2° is used, but no high cut-off angle is used, as all the boundaries in the volume are dislocation boundaries. All 26 nearest-neighbor voxels are included in the calculation of the KAM values. The result is shown in Fig. 4, showing that nucleation takes place preferentially in areas of high stored energy.

The present data also allows a detailed analysis of why the embryonic volumes continue to grow in the deformed microstructure – i.e. grow to become the nuclei. It is generally reported that high angle boundaries (e.g. >10°) migrate faster than low angle boundaries^2^,^15^. It is thus of interest to use the available 4D data (3D plus time) to analyze the fraction of high angle boundaries surrounding the 9 nuclei. Looking first at the misorientations between the voxels identified as the embryonic volume for each nucleus and the voxels in the surrounding volume, it is found that 46% of the boundary segments surrounding the embryonic volumes are high angle boundaries. The corresponding fraction for 9 sites of the same size chosen at random locations in the deformed matrix is less than 1%.

**Figure 5:**
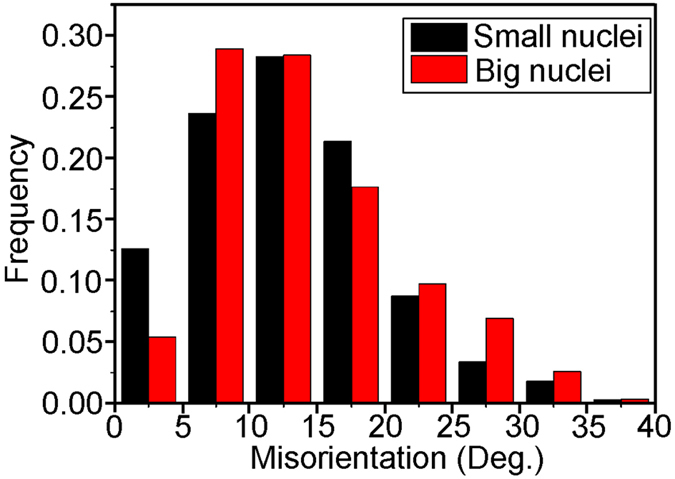
The distribution of misorientations between nuclei and the deformed matrix consumed by the nuclei. The misorientations for the two largest nuclei are shown in red and in black for the three smallest nuclei.

During further continued growth from the initial nucleation sites, the nuclei continue to be partly surrounded by high angle boundaries. The misorientations between the nuclei and the deformed matrix “consumed” by the nuclei are analyzed for the 2 largest and the 3 smallest nuclei (see Fig. 5). It is found that for both groups more than 63% of the misorientations are above 10°, while 13% and 5% are below 5° for the 3 smallest and the 2 largest nuclei, respectively. The nuclei that have grown the least in the present experiments thus have more than twice the frequency of boundaries with misorientations of 5° or less, i.e. low angle boundaries, which are expected to migrate very slowly^22^.

## Conclusion

The present work highlights the unique capability of the DAXM technique for direct studies of nucleation. For a 12% cold rolled aluminum sample further deformed by a hardness indentation, it was found using this technique that (i) nucleation is stimulated by high stored energy, (ii) the nuclei form with orientations already present in the matrix, and (iii) the viable nuclei are partly surrounded by high angle boundaries (>10°). The experimental data allow the identification of embryonic volumes in the deformed microstructure. It is suggested that dislocation cells inside such volumes that are significantly misoriented from neighboring deformed material are the potential nucleation sites. The nucleation mechanism may thus be strain induced *dislocation* boundary migration. The results further indicate that boundaries between nuclei and the deformed matrix of misorientation angle less than 5° hinder their growth.

In our view, experiments of the present type demonstrate the possibilities for obtaining direct information on active nucleation sites and nucleation mechanisms - information absolutely necessary to advance the understanding and modelling of nucleation of recrystallization. The next steps will be to investigate nucleation in more complex, yet still simple samples, with some control of preferential nucleation sites, such as samples with large second phase particles and columnar grained samples with long well defined triple junction lines. The ultimate goal for this type of investigation is to study the most complex situation, namely nucleation in deformed single phase polycrystalline samples.

## Methods

Pure (99.996%) aluminum was chosen for the present study. A starting material with large grains (500 μm–7 mm) was prepared by annealing at 550 °C for 24 h. The starting material was then cold rolled to 12% reduction in thickness, after which a sample of size 6.0 × 4.0 × 1.3 mm^3^ was mechanically cut and the plane formed by the rolling direction (RD) and transverse direction (TD) was electro polished to remove surface damage. Hardness indentations were made on the RD-TD plane with a Vickers diamond indenter of pyramidal shape with a square base and an angle of 136° between opposite faces using a load of 500 g. A previous investigation using exactly these experimental conditions has shown that nucleation preferentially occurs near hardness indentations in grains with higher average hardness^17^. For the present experiment a large grain with orientation {3-1-1} <215>, proven to have high nucleation probability, was chosen. Within this grain, the indentation with the highest hardness of 27.3 Kgf/mm^2^ was selected, to facilitate observation of nucleation upon subsequent annealing.

The microstructure around the indentation tip was mapped using white-beam differential-aperture X-ray microscopy (DAXM), also called the X-ray microbeam Laue diffraction technique, at beam line 34-ID-E at the Advanced Photon Source (APS) in Argonne National Laboratory, USA^23^. For the DAXM experiments, a focused polychromatic X-ray microbeam with a Lorentzian profile and a full-width half maximum of ≈0.5 μm was produced using two non-dispersive Kirkpatrick-Baez (K-B) mirrors. The sample was mounted on a sample holder at a 45° incidence angle to the incident focused microbeam. The Laue diffraction pattern from the whole volume illuminated by the incident microbeam was recorded on a Perkin-Elmer flat panel detector mounted in a 90° reflection geometry, 510.3 mm above the sample. To resolve the diffraction pattern from each volume element (e.g. a subgrain) at different depths, a Pt-wire of 50 μm diameter was used as a differential aperture and scanned at a distance of ≈100 μm from the sample surface. The Laue patterns at each depth were reconstructed by ray-tracing using the LaueGo software available at APS beamline 34-ID-E^24^. Reconstructions of diffraction patterns from individual voxels were conducted to a depth of about 240 μm into the sample with a step size of 1.5 μm. The crystallographic orientations were then indexed based on the depth-resolved Laue patterns (see an example in the supplementary Fig. S2). The orientation resolution obtained with this technique is about 0.01°. By scanning the focused microbeam horizontally and vertically with step size of 1.5 μm and repeating the wire scan at each position, a volume of 243 × 79.5 × 76.5 μm^3^ around the indentation tip was mapped (see Fig. 1).

The sample was then annealed *ex-situ* at 275 °C for 10 min in an air furnace to stimulate nucleation, and then characterized again. Finding the exact same volume as that characterized before annealing was facilitated by use of three Pt fiducial marks which were deposited using a focused ion beam instrument prior to the experiment, placed about 160 μm away from the center of the indentation. By matching the microstructure and shape of the hardness indent in the remeasured volume before and after remounting, a further refinement to give 1.5 μm positional accuracy was achieved.

## Additional Information

**How to cite this article**: Xu, C. *et al*. Direct observation of nucleation in the bulk of an opaque sample. *Sci. Rep.* **7**, 42508; doi: 10.1038/srep42508 (2017).

**Publisher's note:** Springer Nature remains neutral with regard to jurisdictional claims in published maps and institutional affiliations.

## Supplementary Material

Supplementary Material
