# Supplementary material for: Direct observation of nucleation in the bulk of an opaque sample
Source: Sci Rep. 2017 Feb 14;7:42508. doi: 10.1038/srep42508 (PMC5307335; doi:10.1038/srep42508)
Supplement: Supplementary Material [file srep42508-s1.pdf]

Direct observation of nucleation in the bulk of an opaque sample

CHAOLING XU[1, 2], YUBIN ZHANG[1], ANDREW GODFREY[3], GUILIN WU[2*], WENJUN LIU[4], JONATHAN Z. TISCHLER[4], QING LIU[2], DORTE JUUL JENSEN[1*]

[1] Section for Materials Science and Advanced Characterization, Department of Wind Energy, Technical University of Denmark

[2] School of Materials Science and Engineering, Chongqing University, Chongqing 400044, China

[3] Key Laboratory of Advanced Materials (MoE), School of Material Science and Engineering, Tsinghua University, Beijing 100084, P.R. China

[4] Advanced Photon Source, Argonne National Laboratory, Argonne, Illinois 60439-4800, USA

*email: doje@dtu.dk, wugl@cqu.edu.cn

Supplementary Figure S1:

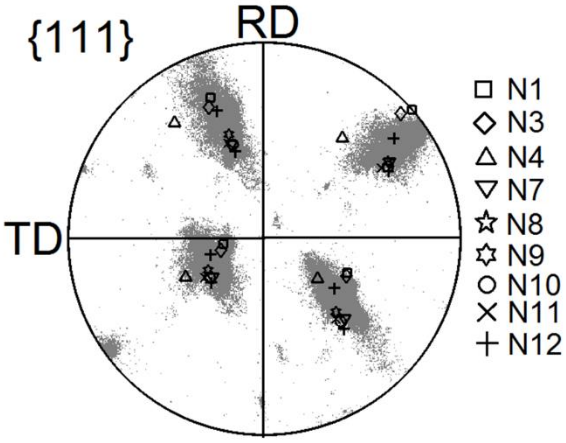

Figure S1: {111} pole figure showing the orientation relationship between the 9 nuclei and the whole of the deformed matrix measured before annealing. The grey cloud represents the orientation distribution of the whole deformed matrix and the other markers indicate the nuclei orientations, labelled as shown in the legend.

Supplementary Figure S2:

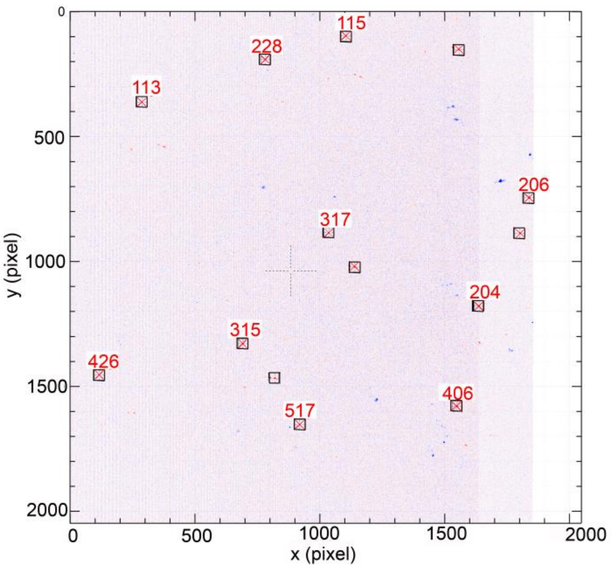

Figure S2: An example depth-resolved Laue diffraction pattern from a voxel-sized deformed volume 75 µm below the sample surface. The detected diffraction spots are marked by squares. Spots that are indexed are indicated by the hkl indexes of the identified diffracting crystallographic planes.
